# The profile of patients with obstructive uropathy in Cameroon: case of the Douala General Hospital

**DOI:** 10.11604/pamj.2016.23.67.8170

**Published:** 2016-03-03

**Authors:** Marie Patrice Halle, Linda Njonkam Toukep, Samuel Ekane Nzuobontane, Hermine Fouda Ebana, Gregory Halle Ekane, Eugene Belley Priso

**Affiliations:** 1 Faculty of Medicine and Pharmaceutical Sciences, University of Douala, Douala, Cameroon; 2 Douala General Hospital, Douala, Cameroon; 3 Faculty of Health Sciences, University of Buea, Buea, Cameroon; 4 Faculty of Medicine and Biomedical Sciences, University of Yaoundé, Yaoundé, Cameroon

**Keywords:** Profile, outcome, obstructive uropathy, Douala general hospital, Cameroon

## Abstract

**Introduction:**

Obstructive uropathy can lead to irreversible kidney damage. The etiology largely determined by the patient's age can be benign or malignant. This study aimed at determining the profile and outcome of patients with obstructive uropathy in Cameroon.

**Methods:**

A cross sectional study carried out in the urology unit of the Douala General Hospital, including patients with a diagnosis of obstructive uropathy seen from January 2004 to December 2013. Clinical profile, treatment and outcome data were obtained from patients records.

**Results:**

Of the 229 patients included 69% were men, mean age 50 ±18 years. Associated comorbidities were hypertension, diabetes, and HIV. Mean haemoglobin 8,40±2,4g/dl, mean GFR 10,3 ±10ml/min, 94 (41%) patients needed emergency dialysis. Symptoms at presentation: asthenia (57%), anorexia (55%), loin pain (37%), vomiting (28%), oedema (20%), and anuria (15%). Urinary tract infection was present in 33 patients. Main aetiologies of obstruction: urolithiasis (35%), begnin prostatic hypertrophy (27%), prostatic cancer (12%), cervical cancer (16%), and congenital malformations (5%). Drainage was effective in 102 (45%) patients, 63 (28%) recovered completely, 91 (41%) were loss to follow up, 49 (22%) died and more women (p = 0.02). Mortality was associated with prostatic cancer (p = 0.000), cervical cancer (p = 0.004) and radiotherapy (p = 0.03).

**Conclusion:**

Patients with obstructive uropathy presented with significant impaired renal function. Main causes were urinary stones, prostatic hypertrophy, prostatic and cervical cancers. Renal recovery was poor, loss to follow up and mortality high. Specific strategies to target improvement in renal recovery and patient's survival are needed in this patient's group.

## Introduction

Obstructive nephropathy, a relatively common condition for practicing urologists refers to the mechanical or functional changes in the urinary tract that interfere with normal urinary flow. [1]. It may be acute or chronic, complete or incomplete, unilateral or bilateral and can lead to rapid deterioration in renal function and irreversible kidney damageif urinary drainage is not rapidly corrected [2, 3]. Obstruction is a relatively common cause of community-acquired akut kidney injury [4, 5]. In a study carried out in Sudan 40% of participants with obstructive uropathy presented with significant renal impairment and 23% needed emergency dialysis [6]. The etiologies are diverse, can be benign or malignant, largely determined by the age of the patient. In children the main aetiologies are uretero-pelvic junction obstruction and congenital urethral valves and meatal stenosis [6–8]. In young adults, calculi are primary cause while in older patients benign prostatic hyperplasia, calculi and malignancy are the common causes [1, 6]. Hydro nephrosis is a usual situation in the course of advanced malignancies(cervical, bladder, prostate, or colorectal cancer)in adults and the cause of obstruction may be invasive-infiltration of the ureters by tumor extrinsic compression by a retroperitoneal primary or metastatic neoplasia, and this may be aggravated by periureteral fibrosis, secondary to previous chemotherapy and radiation therapy [9, 10]. The signs of obstructive nephropathy are often nonspecific and variable depending on the time interval over which the obstruction occurred, the lateralization and the severity of obstruction. The pattern of clinical presentation can be loin pain, lower urinary tract symptoms, fever, mass effect, urine retention, and anuria, impaired renal function with uremic signs [6, 8, 11]. Regardless of the patient′s age, appropriate diagnosis and prompt surgical or interven¬tional drainageis necessary to avoid irreversible renal damage [12]. It is often-reversible and the degree of renal recovery depends primarily on the extent and duration of the obstruction together with the presence or absence of other comorbidity [13]. In a study on patients with obstructive uropathy in Sudan renal function recovery was 100% in patients with acute obstruction and was stabilized in 90% of patients with chronic obstruction and 4 patients had end-stage renal failure [6]. In case of malignancy, the prognosis is often poor and studies have shown that malignancy is a factor of increase morbidity and mortality [14]. Most publications on the topic have focused on individual causes of obstruction or obstruction in specific populations. Few data on the general profile and outcome of patients with obstructive uropathy exist in Sub-Saharan Africa (SSA). We therefore in this study determined the patterns of presentation, the causes, management and outcome of patients with obstructive uropathy in a tertiary referral hospital in Cameroon, with the aim to improve the knowledge and care of this patients in SSA.

## Methods

### Study setting

This was a cross sectional study carried out in the urology unit of the Douala General Hospital (DGH) in Cameroon a tertiary public hospital and one of the main reference hospital in the country. It has the unique public haemodialysis centre of the littoral region (approximately 3millions inhabitants) and the largest of the country and serves as the referral hospital for most patients with kidney and urologic diseases in the region. Ethical approval was obtained from the Douala University Ethical review board, and administrative authorization from the DGH.

### Patients and methods

Medical files of patients with a confirmed diagnosis of obstructive uropathy seen in the urologic units from January 1, 2004 to December 31, 2013 irrespective from age were included. For all patients the following data were collected by a final year's undergraduate medical student: socio-demographic including age (in years), sex. Clinical data such as major comorbidities (hypertension, diabetes, HIV, gout,) signs and aetiology of obstruction, urine output, biological variables (haemoglobin, urea and creatinine, urine dipstick and culture), therapeutic aspect and outcome were recorded. The diagnosis of obstructive uropathy was based on unilateral or bilateral ureteropelvic dilatation confirmed by ultrasonography and/or computerized tomography. Kidney failure was all patients with elevated serum creatinine and estimated glomerular filtration rate (eGFR) <60ml: min/ 1.73m^2^ on admission.

### Statistical analysis

Data were analysed using the software STATA, version 11.1 Results were presented as count and percentages, mean and standard deviation (SD). The comparison of the qualitative variables was made with the Chi-squared test and the quantitative variables with the student testand the Kolmogorov-Smirnov test was used for dichotomic variables. P-values of < 0.05 were considered signifi¬cant. Logistic regression was used to determine the risk factors associated with mortality.

## Results

Two third of the 229 patients included were men. Mean age was 50 ±18 years. Associated comorbidities were hypertension, diabetes, HIV and gout. Main symptoms at presentation were asthenia (57%), anorexia (55%), loin pain (37%), vomiting (28%), oedema (20%), dyspnoea (10%), oliguria (33%), anuria (15%), and macroscopic haematuria (7%). Urinary tract infection was present in 33 (15%) patients (Table 1). Mean haemoglobin was 8,40 ± 2,4g/dl and mean GFR 10,3 ± 10ml/min/ 1.73m2. Renal function impairment occur in 172 (76%) patients, with 94 (41%) of emergency dialysis need (Table 1). Main aetiologies of obstruction were urolithiasis (35%), begnin prostatic hypertrophy (27%), prostatic cancer (12%), cervical cancer (16%), congenital urethral valves and pelvi ureteral junction obstruction (5%), (Table 2). Drainage was done in 102 (45%) patients, mainly bladder catheterization (19.6%) (p = 0.005) and double JJ insertion (19%), Adjuvants treatment were analgesics, chemotherapy, radiotherapy more in women (p = 0.007), hormonal therapy (p= 0.03), and Alfa-blocker (p= 0.001) more used in men (Table 3). Complete renal recovery occurred in 63 (28%) of patients, 94 (41%) were lost to follow up and 49 (22%) patients died and this was higher amongst women (p = 0.02). Prostatic cancer (p = 0.000), cervical cancer (0.004) and radiotherapy (0.03) were factors associated with mortality (Table 4).

**Table 1:** General characteristics of patients with obstructive uropathy

Variables	Total N = 226	Male(%) N = 156(69)	Female (%) N = 70(31)	P
Mean age (years±SD)	50±18	50,7±20	48,6±14	0.43
Hypertension	39(17)	30(19)	9(13)	0.24
Diabetes Mellitus	13(6)	11(7)	2(3)	0.21
Gout	4(2)	4(3)	(0)	0.17
Asthenia	128(57)	85(54)	43(61)	0.30
Anorexia	124(55)	82(53)	42(60)	0.29
Hiccup	28(12)	18(12)	10(14)	0.56
Vomiting	64(28)	37(24)	27(39)	**0.02**
Insomnia	39(17)	25(16)	14(20)	0.40
Cramps	30(13)	20(13)	10(14)	0.70
Lower limb Oedema	46(20)	25(16)	21(30)	**0.01**
Dyspnea	23(10)	13(8)	10(14)	0.10
Dysuria	33(15)	25(16)	8(11)	0.50
Abdominal pain	84(37)	54(35)	30(43)	0.78
Fever	18(8)	10 (6)	8(11)	0.10
Macroscopichematuria	15(7)	9(6)	6(6)	0.43
Urinary tract infection	33(15)	25(16)	8(11)	0.50
Anuria	33(15)	15(10)	18(26)	**0.001**
Oliguria	74(33)	59(38)	15(21)	**0.01**

**Table 2:** Biological parameters and etiologies of obstructive uropathy

Variables	Total	Male(%)	Female(%)	P
Mean Heamoglobin (SD)	8.40 (±2.4)	8.5 (±2.2)	8.1 (±2.8)	0.31
Mean Urea (SD)	1.27(±1.07)	1.25 (±1.07)	1.31 (±1.07)	0.71
Mean Creatinine (SD)	79.34 (±86)	75.9 (±89)	84.6 (±82)	0.56
Mean GFR(SD)	10.3(±10.3)	11.6 (±10)	7.7 (±10)	0.06
Impaired renal fonction	172(76)	121(77.5)	51(73)	0.44
Bladder Cancer	10(4)	7(5)	3(4)	0.70
Congenital malformations	12(5)	11(7)	1(1)	0.08
Retroperitoneal fibrosis	3(1)	1(0.6)	2(3)	0.17

**Table 3:** Treatment and outcome of patients with obstructive uropathy

Variables	Total	Male(%)	Female(%)	P
Urethral catherization	44(19.6)	28(24)	6(9)	**0.005**
Double JJ	43(19)	27(17)	16(23)	0.32
Suprapubic catherization	14(6)	12(8)	2(3)	0.16
Nephrostomy	1(0.4)	0(0)	1(1.4)	0.13
Indication for Dialysis	94(41)	62(40)	32(46)	0.20
Dialysis done	41(18)	31(20)	10(14)	0.12
Chemotherapy	20 (9)	3(2)	17(24)	0.07
Radiotherapy	12 (5)	3(2)	9(13)	**0.007**
Hormonotherapy	18(8)	18(12)	0(0)	**0.003**
Alpha blockers	21(9)	21(13)	0(0)	**0.001**
Analgesic	86(39)	58(37)	28(40)	0.094
Total renal recovery	63(28)	49(31)	14(20)	0.07
Partial renal recovery	8(4)	7(4)	1(1)	0.10
No recovery	12(5)	10(6)	2(3)	0.14
Lost to follow up	94(41)	69(44)	29(41)	0.69
Death	49(22)	25(16)	24(34)	**0.002**

**Table 4:** Factors associated to mortality of patients with obstructive uropathy

Factors			Univariate analysis		Multivariate analysis	
	No Death (N = 177)	Death(N = 49)	OR (95% CI)	*P*	OR (95% CI)	P
Age ≥ 50 ans	93	33	2.6(0.8-8.2)	0.09	1.1(0.25-4)	0.89
Female	46	24	2.7(1.4-5.3)	0.003	1.25(0.17-9)	0.8
Male	131	25	0.36 (0.1-0.7)	0.001		
Fever	11	7	2.5(0.9-6.8)	0.07		
Uremic syndrome	53	39	9.1 (4.2-19.6)	0.000	1.3(0.5-8)	0.26
Fluid overload	18	20	6.1 (2.8-12.8)	0.000	1.3(0.4-3)	0.5
Anuria	15	18	6.2(2.9-13.7)	0.000	2.2(0.6-7)	0.18
Urinary tract infection	31	2	0.75(0.1-4.1)	0.7		
prostatic cancer	12	14	23.3(5.7-93.9)	0.000	25(4-149)	**0.000**
Cervical Cancer	14	21	8.7 (3.7-19.1)	0.000	1.7(2-8.5)	**0.004**
Absence of Dialysis	147	38	1.4(0.6-3)	0.3	0.3(0.8-1.1)	0.07
Radiotherapy	2	10	22.4 (4.7106.4)	0.000	10(1.7-97)	**0.03**
Chemotherapy	8	12	6.8 (2.6-17.9)	0.000	1.2 (0.2-5)	0.8

## Discussion

This study on the clinical profile and outcome of patients with obstructive uropathy treated in a tertiary hospital in Cameroon is the first one in our setting. It revealed that ¾ of participants had renal impairment at presentation with various symptoms (uremic, anuria and overload) and 41% needed emergency dialysis but only half of them could benefit from this treatment. Hypertension, diabetes and HIV were the main associated comorbidities. Fifteen percent of patients had urinary tract infection. The main aetiologies of obstruction were urolithiasis, begnin prostatic hypertrophy, cancer mainly prostatic and cervical cancer, and congenital malformations. Drainage was done only in 45% of patients. Renal recovery occurred was low and almost half of patients were loss of follow up. 1/5 patients died and mortality was associated with prostatic, and cervical malignancies and radiotherapy. Urinary tract obstruction is a common clinical problem facing urologist. It may be acute or chronic, partial or complete, unilateral or bilateral, can occur at any site of the urinary tract and lead to rapid deterioration in renal function and irreversible kidney damage if urinary drainage is not corrected in a time [7]. In our study 76% of patients had renal function impairment at presentation with 41% requiring emergency dialysis. These results are consistent with the literature: obstruction is a relatively common cause of kidney failure but the rate of severe renal insufficient with a need for emergency dialysis in our study was higher compared to a similar study by Iman in Sudan where 40% presented with significant renal impairment and 23% required emergency dialysis [4–6]. Certain patient-specific factors especially CKD traditional risk factors may increase the risk of kidney function deterioration amongst patients with obstruction. In this study, associated comorbidities such as hypertension, diabetes and HIV and also urinary tract infections were frequent, and this could probably contribute to the deterioration of renal function of these patients.

Signs of obstructive nephropathy are often non-specific, depending on the time interval over which the obstruction occurs, the lateralization and the severity of obstruction. Although a decrease in urine output is frequently observed, normal or elevated urine output does not exclude partial obstruction. In the present study 15% of patients had anuria and 33% oliguria. However, urine output was normal in almost half of patients. Patients presented with various symptoms especially uremic sign, sign of volume over load and loin pain. The explanation is the late presentation of patients with severe renal function deterioration and also by the aetiology of the obstruction that was mainly urolithiasis and cancer in this study. These findings are consistent with others studies [6, 8, 11]. Urolithiasis was the main begnin aetiology (35%) of obstruction in this serie and this rate is very high compared to others studies [11, 14]. BPH was the second cause of obstruction in our study. BHP is a problem experienced by aging men and is the most common benign aetiology of obstruction in men, our result are similar to the literature were BHP accounted for 30% of obstruction in one serie [15]. Hydronephrosis is a common situation in cases of advanced malignancies, and the cause of obstruction may be invasion of the ureters by tumor, extrinsic compression by a retroperitoneal primary or metastatic neoplasia. Cancer was the cause of obstruction in 32% of our participants. This rate is very high compare to the study of El Iman in Soudan where cancer accounted only for 8% of cases [6]. The difference could be due to the fact that our study was done in a tertiary referral hospital where patients with malignancy in the region are usually referred late. Cervical cancer in women and prostatic cancer in men were the leading malignancy in our serie. Due to the proximity of the cervix to the bladder neck, obstruction can complicate 30% of cervical cancers [16] and despite advances in early detection of prostate cancer, 10% of patients presented with locally advanced prostate cancer with upper urinary tract obstruction as their main symptoms [17].

Once the diagnosis of obstructive uropathy is made, prompt and appropriate intervention is necessary to avoid irreversible renal damage. Active surgical intervention and creation of adequate urine outflow from the obstructed kidney is the method of choice for initial treatment even in case of malignancy irrespective of the disease stage [10, 18]. In our study drainage could be done only in 45% of participants and this concerned mainly patients without malignancy. Some reasons for this low rate of drainage procedure could be the financial constraint in a setting where health insurance is almost inexistent with a high rate of patients who were lost to follow up after the first consultation, the lack of appropriate material and especially the late stage at presentation of patients with malignancy. Even if others studies have shown that bypassing the obstruction in case of malignancy is a successful way to prolong life, most of our patients did not benefit from it [19, 20]. The degree of renal recovery depends first on the extent and duration of the obstruction together with the presence or absence of infection [11]. Total renal recovery occurred in 28% of cases in this study and was partial in 4%. Renal outcome was undetermined in almost half of the participants who were lost to follow up. The number of patients who were lost to follow up is very high in this study and mainly due to financial constraint and the ignorance of the consequences of the disease. The mortality rate of 22% was associated to cervical and prostatic cancer, and radiotherapy. Based on our observations, patients with bilateral obstruction secondary to malignant cancer should be counselled that their prognosis is poor. These results are consistent with other reports in which a malignant cause of obstructive uropathy is considered as a prognostic indicator of morbidity and reduced survival [14, 21–23].

### Limitations

This study has some limitations. The retrospective data collection from hospital files may induce some inaccuracies and missing data. Also it was a single centre study so the findings may not be generalized. However, this study is the first to describe the profile and outcomes of patients with obstructive uropathy in our setting with a heterogeneous group, in a referral tertiary hospital. It therefore provides background data that will contribute to raise awareness and enhance further research in this domain.

## Conclusion

Patients with obstructive uropathy in our setting presented with significant impaired renal function Urinary stones and BPH are the common begnin causes while prostatic and cervical cancer account for the majority of malignancy. Renal recovery is poor, loss to follow up and mortality especially due to cancer is high. Specific strategies to target improvements in renal recov¬ery and patient's survival are needed in this patient group.

### What is known about this topic

- Obstructive nephropathy, is a relatively common condition and the main aetiologies depending on the age of patients congenital malformations, calculi and tumor.
- It can lead to renal failure and it is mostly reversible if medical and surgical treatment is done early.
- Mortality is usually very low.

### What this study adds

- Calculi and malignancies were the mains aetiologies.
- Patients presented late, with renal failure in 76% of cases and need for emergency dialysis.
- Complete renal recovery occurred in 28% of case and 41% of patients were lost of follow up and 22% died mainly due to malignancies.
